# Qianggu capsule for the treatment of primary osteoporosis: evidence from a Chinese patent medicine

**DOI:** 10.1186/s12906-017-1617-3

**Published:** 2017-02-13

**Authors:** Xu Wei, Aili Xu, Hao Shen, Yanming Xie

**Affiliations:** 1 Department of Scientific Research, Wangjing Hospital, China Academy of Chinese Medical Sciences, Beijing, 100102 China; 2 Institute of Orthopaedics of Beijing Integrative Medicine, Beijing, 100700 China; 3 Department of Gastroenterology, Wangjing Hospital, China Academy of Chinese Medical Sciences, Beijing, 100102 China; 4 Institute of Basic Research in Clinical Medicine, China Academy of Chinese Medical Sciences, Beijing, 100700 China

**Keywords:** Qianggu Capsule, Primary osteoporosis, Chinese patent medicine, Systematic review

## Abstract

**Background:**

Qianggu Capsule, a Chinese patent medicine, has been widely applied in the clinical practice of primary osteoporosis (POP) in recent years. This study aims to summarize the effectiveness and safety of Qianggu Capsule in treating POP.

**Methods:**

We searched seven electronic databases, all searches ended in 30 September, 2015. All randomised controlled trials comparing the efficacy of Qianggu Capsule treatment with no treatment, placebo or conventional therapy for POP were included. Combined therapies of Qianggu Capsule were also included. Cochrane risk of bias tool was used to assess methodological quality of primary studies. Revman 5.2.0 software was used for data analysis.

**Results:**

Ten trials were enrolled. The combined effect showed that Qianggu Capsule plus Caltrate D was better than Caltrate D on lumbar spine bone mineral density (BMD) (MD = 0.05 g/cm^2^; 95% CI: 0.02–0.07; *P* = 0.0004), femoral neck BMD (MD = 0.03 g/cm^2^; 95% CI: 0.01–0.05; *P* = 0.001), femoral great trochanter BMD (MD = 0.04 g/cm^2^; 95% CI: 0.03–0.06; *P* < 0.001). Meta-analysis exhibited a significant antiosteoporosis effect of Qianggu Capsule on femoral neck BMD (MD = 0.03 g/cm^2^; 95% CI: 0.01–0.05; *P* = 0.003) and femoral trochanteric BMD (MD = 0.07 g/cm^2^; 95% CI: 0.02–0.12; *P* = 0.006) compared with α-D3 capsule. However, the methodological quality of included studies was low. Constipation and dry mouth were the most common adverse drug reactions of Qianggu Capsule. Finally the evidence level was evaluated to be low or very low.

**Conclusions:**

The effect of Qianggu Capsule for POP was supported in improving BMD. Due to the methodological drawbacks of the included studies, the conclusions should be treated with caution for future research.

## Background

Primary osteoporosis (POP) is one of the most common chronic conditions, and affects both old men and postmenopausal women [1, 2]. Osteoporosis is estimated to cause 1.5 million fractures every year in the United States [3]. In China, there have been about 202.43 million people aged 60 years and older at the end of 2013, which faces higher risk of osteoporosis-related fractures [4]. From 2002 to 2006, the rates of hip fracture over age 50 years have increased 58% in women and 49% in men based on a population-based study in Beijing [5]. Most important of all, the most serious consequences of osteoporotic fractures, especially hip fracture, are the increasing proportion of mortality and disability [6]. Therefore, interventions to treat POP or prevent osteoporotic fractures should be implemented. Although research efforts have been expanded for several decades, an urgent need exists for continued improvement so far, particularly in the treatment of POP.

Many strategies are available to treat POP, but pharmacological treatments still plays the dominant role. Major antiosteoporosis agents including bisphosphonates, denosumab, hormone replacement therapy, selective estrogen receptor modulators, recombinant human parathyroid hormone and strontium ranelate are currently available on the market [7]. The common outcomes are osteoporotic fractures [8, 9], bone mineral density (BMD) value [10], bone turnover markers [11], pain assessment [12], quality of life [13], and adverse event or adverse drug reaction mainly from antiosteoporosis drugs [14]. In some cases, POP patients can benefit from drug therapy optimization and combination therapy. Despite the fact that several western medicines have demonstrated to be effective in the treatment of POP, however, poor medication adherence remains a major problem [15, 16]. Suboptimal adherence to therapy may partially be due to adverse effects of long-term conventional antiosteoporosis drugs, such as bisphosphonates [17, 18]. Hence, there is a requirement for long-term treatment to be associated with a positive benefit-risk balance [19]. Now more and more studies of complementary and alternative medicine have increased the awareness of the problem and have improved our understanding of the prevention and control of osteoporosis. In China, herbal fufang and single Chinese herb have been widely used for the treatment of POP [20–22].

Qianggu Capsule, the main effective components of which are the total flavonoids of *Rhizoma Drynariae* (Gusuibu) [23], has been approved by China Food and Drug Administration for treating POP (drug approval numbers: Z20030007). According to the theory of traditional Chinese medicine and results of population pharmacokinetics, Qianggu Capsule has the effect of replenishing the kidney and strengthening the bones which applies to shen-yang deficiency pattern [24, 25]. Modern research has also proven that Qianggu Capsule can increase lumbar and femoral BMD, raise serum calcium, improve analgesia action, control the levels of serum IL-6 and TNFa, and accelerate the secretion of IL-4 in rats. No abnormal changes are found in the toxicity test [26]. So Qianggu Capsule is reliable and safe in laboratory studies.

In contrast to the wealth of data about the efficacy of chemical agents in the management of POP, information regarding their efficacy and safety in Chinese herbal medicine is relatively limited. In recent years, a large number of clinical studies reported the effect of Qianggu Capsule and Qianggu Capsule combined with antiosteoporosis drugs. Therefore, this systematic review provides an evidence of Qianggu Capsule for the management of POP from the randomised controlled trials.

## Methods

The study protocol was previously registered in PROSPERO platform which could be available on https://www.crd.york.ac.uk/PROSPERO/display_record.asp?ID=CRD42015025784.

### Data sources and searches

Seven electronic databases were searched from their inception until 30 September, 2015: PubMed, Cochrane CENTRAL, EMBASE, Chinese National Knowledge Infrastructure (CNKI), Wanfang database, Chinese Scientific Journals Database (VIP), Chinese Biomedical Literature Database (CBM). Additional published or unpublished literature was retrieved through manual searches of reference lists of included studies and key review articles, and from the files of content experts.

The search terms included “osteoporosis”, “primary osteoporosis”, “senile osteoporosis”, “postmenopausal osteoporosis”, “qianggu capsule”, “qiang gu capsule” and “Gusuibu”. Search terms used for PubMed were as follows: (osteoporosis OR primary osteoporosis OR senile osteoporosis OR postmenopausal osteoporosis) AND (qianggu capsule OR qiang gu capsule OR Gusuibu).

### Types of studies

All completed randomised controlled trials comparing the efficacy of Qianggu Capsule treatment for POP were enrolled. Animal experiments were not inclusive.

### Types of participants

The clinical diagnosis was required to be in accordance with the criteria of POP. It should be noted that some minor differences existed among different diagnostic criteria. For example, World Health Organization criteria (BMD of subjects, 2.5 SD [T-score < or = −2.5] lower than young adult mean value) [27] had a different numerical standard than that for Chinese criteria (BMD of subjects, 2 SD [T-score < or = −2] or less than 75% of lower than young adult mean value) [28, 29]. Generally, study population was mainly from middle-aged and aged people (≥40 years).

### Types of interventions

In this review, randomised controlled trials that assessed the therapeutic effect of Qianggu Capsule, compared with no treatment, placebo or conventional therapy were considered. Combined therapies of Qianggu Capsule and other conventional interventions compared with other conventional interventions in randomised controlled trials were also enrolled. The interventions containing other complementary and alternative treatments (Chinese medicine, acupuncture, moxibustion, massage, yoga, tai chi, qigong, baduanjin, wuqinxi and so on) in the Qianggu Capsule or comparison group were excluded. The duration of treatment was required to be at least 3 months.

### Types of outcomes

The primary outcome was osteoporosis-related fractures. The secondary outcomes analyzed in this review were BMD values, pain scores, quality of life, biochemical markers of bone turnover, and adverse event or adverse drug reaction (ADR).

### Study selection

Two reviewers independently searched and screened the studies. Exclusion criteria included: (1) inappropriate study design, such as reviews, case reports, comments, letters; (2) duplicate trials; (3) not population of interest; (4) no Qianggu Capsule intervention; (5) lack of the above outcomes. After removing excluded abstracts, full articles were obtained and studies were screened again more thoroughly using the same exclusion criteria. Any disagreements were resolved through discussion with a third reviewer.

### Data abstraction

Data abstraction was independently performed by two reviewers based on pre-piloted forms. A neutral third reviewer was consulted if there are still disagreements after discussion. The first author names and year of publication, sample size, diagnostic criteria, population characteristics (age and sex), duration of symptom, intervention details (medication doses, therapeutic regimen and treatment duration), and outcome data were extracted.

### Risk of bias assessment in individual studies

We used the Cochrane risk of bias tool to assess methodological quality of included studies [30]. And two authors compared the evaluation results and discussed difference until agreement was reached. Selection bias, performance bias, detection bias, attrition bias, reporting bias and other bias were evaluated respectively. The quality of included trials was divided into three levels: low risk of bias (all the items were in low risk of bias), high risk of bias (at least one item was in high risk of bias), unclear risk of bias (at least one item was in unclear).

### Analytical approach

Data analysis was performed with Review Manager 5.2.0 software. Based on the continuous data, mean difference (MD) was used to assess the difference between experimental group and control group. Standardized mean difference (SMD) was considered if clinical outcome was the same but measured using different scales in the different trials. Risk ratio (RR) was used for the binary data. And the 95% confidence intervals (CI) were calculated in the meta-analysis. In a three-group design study that had two treatment groups of Qianggu Capsule and Qianggu Capsule plus antiosteoporosis drugs, the two comparisons were split in the meta-analysis. Heterogeneity was assessed by means of I^2^ statistic. If the I^2^ statistic indicated considerable heterogeneity (≥50%), we combined the summary measures across the studies using a random effects model that assumed that the included studies represent a sample from a larger population of studies [31]. Analysis of subgroups will be used if there are sufficient clinical trials for the same outcome.

### Qualitative analysis of trial results

We evaluated the quality of the body of evidence adopting the GRADE approach [32, 33]. High quality evidence was considered as randomised controlled trials with low risk of bias that produced consistent, direct and precise results for the clinical outcome [34]. Three domains, including large magnitude of effect, all plausible confounding which can increase confidence in estimated effects, high dose–response gradient may increase the quality of evidence [35, 36]. Levels of quality of evidence were defined as high, moderate, low, very low [37].

## Results

### Characteristics of the studies

**Fig. 1:**
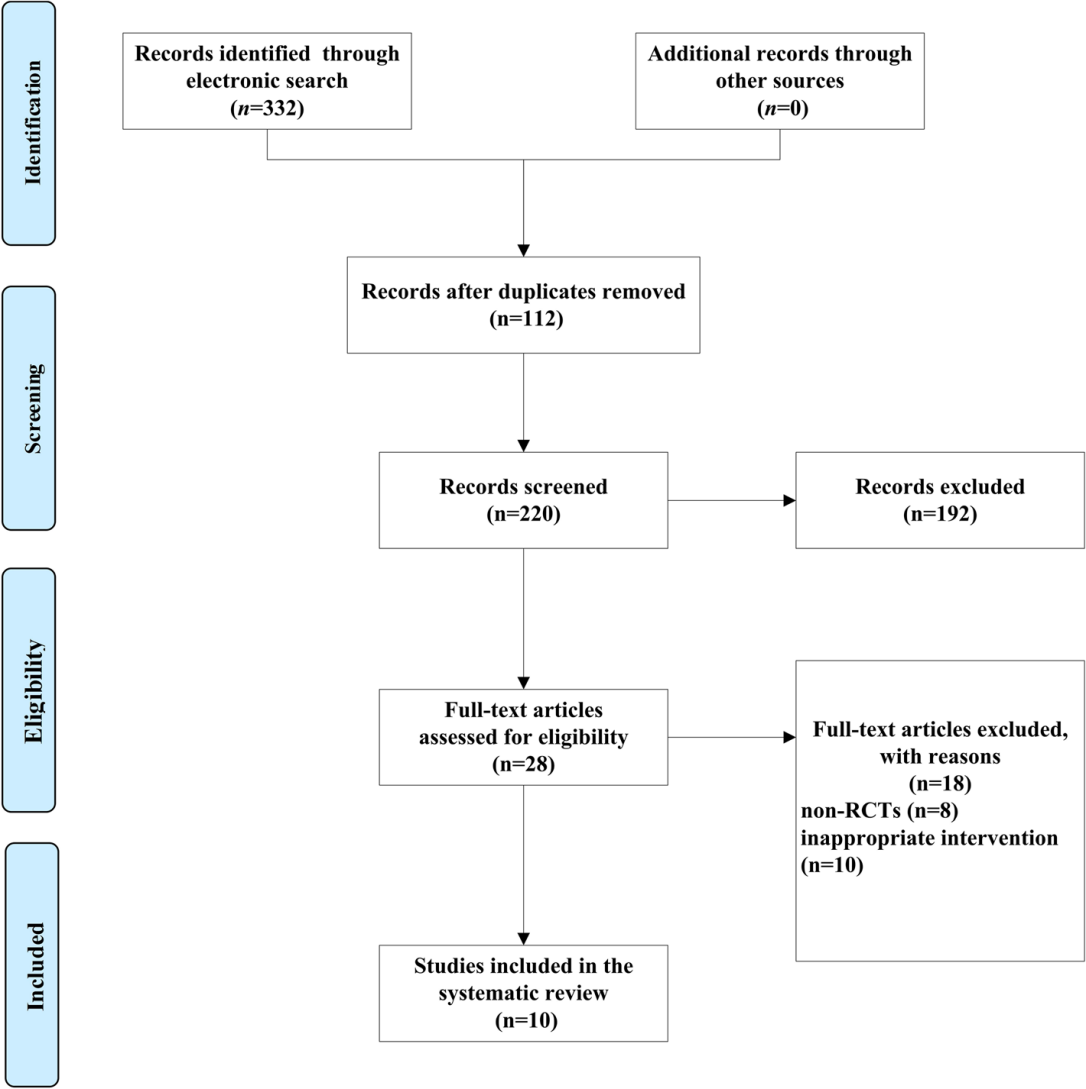
PRISMA flowchart

The search strategy identified 332 reports. After removal of duplicates, 220 records remained. After going through the titles and abstracts, 192 reports were excluded with at least one of following reasons: (1) animal experiments; (2) traditional review or not from POP patients; (3) lack of control group. Then the remaining 28 papers were further assessed with accessible full text. Eventually 10 reports [38–47] met the inclusion criteria for the review and 18 papers were excluded. The reasons for exclusion were: non-RCTs (*n* = 8), inappropriate intervention (*n* = 10). The screening process was showed in a PRISMA 2009 flow diagram (Fig. 1). All the studies were published in Chinese journals (from 2004 to 2013).

Of the 10 articles, 806 participants were enrolled in the review and depicted in Table 1. Eight trials used Chinese osteoporosis diagnostic criteria [38, 40–42, 44–47]. Two trials were also included because BMD was used for the diagnosis and evaluation [39, 43]. The average age ranged from 57.9 to 70.4 years. Course of disease was provided in only 2 trials [41, 47] and was not found in the remaining included studies.

**Table 1:** Characteristics of included trials on Qianggu Capsule for POP

Study ID	Sample size (EG/CG)	Diagnostic criteria	Age (yrs, mean) Sex (male/female)	Course of disease	Experimental group	Comparison group	Duration of treatment	Outcome assessment
Gu and Guo 2004 [38]	82 (41/41)	Chinese criteria	EG: 63.2 (26/15) CG: 62.7 (29/12)	NR	QC (0.25 g, Tid)	Calcium gluconate (3 pills, Tid)	3 months	BMD (LS) ADR
Zhao et al. 2004 [39]	69 (34/35)	NR	EG: NR CG: NR	NR	QC (0.25 g, Tid)	Livial (1.25 mg, Qd)	6 months	BMD (LS、FN) ADR
Xia and Chen 2006 [40]	58 (29/29)	Chinese criteria	EG: 58.6 ± 6.3 (0/29) CG: 57.9 ± 6.7 (0/29)	NR	QC (0.25 g, Tid) + CG	Caltrate D (600 mg, Qd)	12 months	BMD (LS、FN、WA、FGT) ADR
Ji 2006 [41]	62 (40/22)	Chinese criteria	EG: 65.3 (12/28) CG: 65.2 (6/16)	EG: 3.6 years CG: 3.5 years	QC (0.25 g, Tid)	Vitamin D2 and calcium hydrogen phosphate tablets (0.15 g, Tid)	3 months	BMD (ulna、radius)
Shan and Zhou 2006 [42]	62 (32/30)	Chinese criteria	EG: 60.32 ± 4.58 (13/19) CG: 60.96 ± 5.06 (12/18)	NR	QC (0.25 g, Tid)	α-D3 capsule (0.5 μg, Bid)	3 months	BMD (LS、FN、FT) Ca、P、ALP ADR
Wang et al. 2007 [43]	54 (28/26)	NR	EG: 61.8 ± 6.1 (0/28) CG: 62.3 ± 5.9 (0/26)	NR	QC (0.25 g, Tid)	α-D3 capsule (0.5 μg, Bid)	6 months	BMD (LS、FN) Ca、P、ALP、 NTX/Cr ADR
Li and Zhao 2008 [44]	60 (30/30)	Chinese criteria	EG: 61.8 ± 6.1 (0/30) CG: 62.3 ± 5.9 (0/30)	NR	QC (0.25 g, Tid) + Calcium tablet (1 pill, qd)	QC placebo + Calcium tablet (1 pill, qd)	6 months	BMD (LS) BGP、CT、E_2_、PTH、HOP/Cr
Gao 2008 [45]	128 (64/64)	Chinese criteria	EG: 66.23 ± 7.24 (24/40) CG: 65.14 ± 7.51 (26/38)	NR	QC (0.25 g, Tid)	α-D3 capsule (0.5 ~ 1 μg, Bid)	6 months	BMD (LS、FN、WA、FT) ADR
Xu et al. 2010 [46]	80 (40/40)	Chinese criteria	EG: NR (0/40) CG: NR (0/40)	NR	QC (0.25 g, Tid) + CG	Alendronate (70 mg, once a week)	6 months	BMD (LS、WA) ADR
Zeng et al. 2013 [47]	150 (75/75)	Chinese criteria	EG: 70.4 ± 5.8 (39/36) CG: 70.0 ± 5.2 (41/34)	EG: 6.8 years CG: 6.7 years	QC (0.25 g, Tid) + CG	Caltrate D (600 mg, Qd)	12 months	BMD (LS、FN、FGT)

*NOTE*: *EG* experimental group, *CG* comparison group, *NR* not reported, *QC* qianggu capsule, *LS* lumbar spine, *FN* femoral neck, *WA* wards area, *FGT* femoral great trochanter, *FT* femoral trochanteric, *Ca* calcium, *P* phosphorus, *ALP* alkaline phosphatase, *BGP* bone gla protein, *CT* calcitonin, *E*_*2*_ estradiol, *PTH* parathyroid hormone, *NTX* urinary N-telopeptides of type I collagen, *HOP* urinary hydroxyproline, *Cr* Creatinine, *ADR* adverse drug reaction

To reduce the clinical heterogeneity among the studies, the interventions could be divided into 7 different subgroups as follows: (1) Qianggu Capsule versus Calcium gluconate [38]; (2) Qianggu Capsule versus Livial [39]; (3) Qianggu Capsule plus Caltrate D versus Caltrate D [40, 47]; (4) Qianggu Capsule versus Vitamin D2 and calcium hydrogen phosphate tablets [41]; (5) Qianggu Capsule versus α-D3 capsule [42, 43, 45]; (6) Qianggu Capsule and Calcium tablet versus Qianggu Capsule placebo and Calcium tablet [44]; (7) Qianggu Capsule plus Alendronate versus Alendronate [46]. The duration of treatment was not beyond 12 months.

All the studies reported different parts of BMD values [38–47]. Three studies used bone biochemical markers as surrogate outcome [42–44]. Seven studies reported adverse drug reaction (ADR) [38–40, 42, 43, 45, 46]. In addition, osteoporotic fractures, internationally recognized pain scales and quality of life were not evaluated in all trials.

### Quality of methodological reporting

The methodological quality of primary studies was evaluated as low (as shown in Table 2). Only 1 trial reported random number table as the method of randomization [46]. A randomized, double-blind and placebo-controlled trial was identified [44]. Allocation concealment, blinding of participants and personnel were not found in the other studies. The blinding of outcome assessment was not stated in all trials. Two trials did not provide any information about the drop-outs or withdrawals [40, 44]. None of the trials registered or published the study protocol. So the selective reporting was unclear. Additionally, other sources of bias were identified as unclear in 3 trials because the baseline of the trials was not mentioned [38, 41, 44].

**Table 2:** Assessment of methodological quality for randomized controlled trials

Study ID	Random sequence generation	Allocation concealment	Blinding of participants and personnel	Blinding of Outcome assessment	Incomplete outcome data	Selective reporting	Other sources of bias	Risk of bias
Gu and Guo 2004 [38]	Unclear	Unclear	High	Unclear	Low	Unclear	Unclear	High
Zhao et al. 2004 [39]	Unclear	Unclear	High	Unclear	Low	Unclear	Low	High
Xia and Chen 2006 [40]	Unclear	Unclear	High	Unclear	Unclear	Unclear	Low	High
Ji 2006 [41]	Unclear	Unclear	High	Unclear	Low	Unclear	Unclear	High
Shan and Zhou 2006 [42]	Unclear	Unclear	High	Unclear	Low	Unclear	Low	High
Wang et al. 2007 [43]	Unclear	Unclear	High	Unclear	Low	Unclear	Low	High
Li and Zhao 2008 [44]	Unclear	Low	Low	Unclear	Unclear	Unclear	Unclear	Unclear
Gao 2008 [45]	Unclear	Unclear	High	Unclear	Low	Unclear	Low	High
Xu et al. 2010 [46]	Low	Unclear	High	Unclear	Low	Unclear	Low	High
Zeng et al. 2013 [47]	Unclear	Unclear	High	Unclear	Low	Unclear	Low	High

*NOTE*: Unclear: unclear risk of bias; Low: low risk of bias; High: high risk of bias

### Effect of the interventions

All the included studies compared Qianggu Capsule practised alone or combined with antiosteoporosis drugs. According to the different intervention and control program, the interventions could be divided into the following subgroups.

**Fig. 2:**
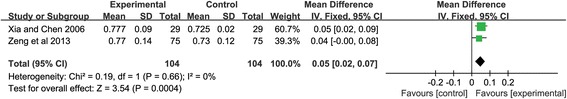
Meta-analysis of Qianggu Capsule plus Caltrate D versus Caltrate D on lumbar spine BMD

**Fig. 3:**
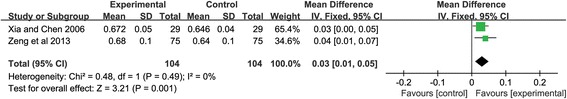
Meta-analysis of Qianggu Capsule plus Caltrate D versus Caltrate D on femoral neck BMD

**Fig. 4:**
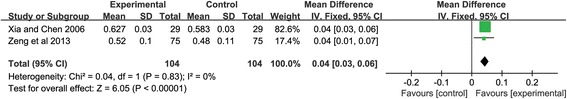
Meta-analysis of Qianggu Capsule plus Caltrate D versus Caltrate D on femoral great trochanter BMD

**Fig. 5:**
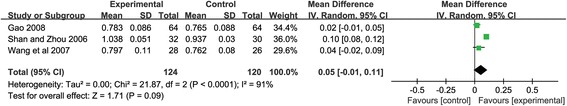
Meta-analysis of Qianggu Capsule versus α-D3 capsule on lumbar spine BMD

**Fig. 6:**
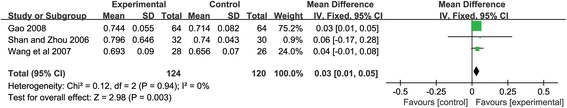
Meta-analysis of Qianggu Capsule versus α-D3 capsule on femoral neck BMD

**Fig. 7:**
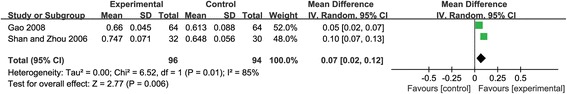
Meta-analysis of Qianggu Capsule versus α-D3 capsule on femoral trochanteric BMD

1. Qianggu Capsule versus Calcium gluconate: there was a statistically significant difference between the groups in mean improvement on lumbar BMD favoring Qianggu Capsule intervention after 3 months (*P* < 0.05) [38].

2. Qianggu Capsule versus Livial: BMD in lumbar spine and femoral neck increased markedly in livial group, but statistical significance was not found in both groups after 6 months (*P* > 0.05) [39].

3. Qianggu Capsule plus Caltrate D versus Caltrate D: The combined analysis of two trials found a significant effect of Qianggu Capsule plus Caltrate D on lumbar spine BMD (MD = 0.05 g/cm^2^; 95% CI: 0.02–0.07; *P* = 0.0004, Fig. 2), femoral neck BMD (MD = 0.03 g/cm^2^; 95% CI: 0.01–0.05; *P* = 0.001, Fig. 3), femoral great trochanter BMD (MD = 0.04 g/cm^2^; 95% CI: 0.03–0.06; *P* < 0.001, Fig. 4) [40, 47]. In addition, there was no significant difference on ward’s BMD between the groups in the result of Xia et al. (*P* < 0.05) [40].

4. Qianggu Capsule versus Vitamin D2 and calcium hydrogen phosphate tablets: Qianggu Capsule group demonstrated a significant improvement on BMD of ulna and radius compared with Vitamin D2 and calcium hydrogen phosphate tablets group after 3 months (*P* < 0.05) [41].

5. Qianggu Capsule versus α-D3 capsule: There was no significant difference on lumbar spine BMD (MD = 0.05 g/cm^2^; 95% CI: −0.01–0.11; *P* = 0.09, Fig. 5) between the groups [42, 43, 45]. Meta-analysis indicated a significant antiosteoporosis effect of Qianggu Capsule on femoral neck BMD (MD = 0.03 g/cm^2^; 95% CI: 0.01–0.05; *P* = 0.003, Fig. 6) [42, 43, 45], femoral trochanteric BMD (MD = 0.07 g/cm^2^; 95% CI: 0.02–0.12; *P* = 0.006, Fig. 7) compared withα-D3 capsule [42, 45]. A remarkable improvement in ward’s BMD with Qianggu Capsule was identified in Gao’s study (*P* < 0.01) [45].

Meta-analysis of two trials showed that there was no difference in improving the level of calcium (MD = 0.01 mmol/L; 95% CI: −0.04–0.06; *P* = 0.69), phosphorus (MD = 0.01 mmol/L; 95% CI: −0.04–0.06; *P* = 0.67) and alkaline phosphatase (MD = 3.05 U/L; 95% CI: −4.66–10.76; *P* = 0.44) [42, 43]. In Wang’s study, Qianggu Capsule was better thanα-D3 capsule in lowering NTX/Cr (*P* < 0.01) [43].

6. Qianggu Capsule and Calcium tablet versus Qianggu Capsule placebo and Calcium tablet: Based on Calcium tablet as basic treatment, Qianggu Capsule was better than placebo in improving lumbar BMD value after 6 months (*P* < 0.01, *P* < 0.05). Qianggu Capsule plus Calcium tablet also significantly increased the level of bone gla protein, calcitonin and estradiol in the blood (*P* < 0.01); on the other, the excretion of urinary hydroxyproline and the level of parathyroid hormone was reduced (*P* < 0.01) [44].

7. Qianggu Capsule plus Alendronate versus Alendronate: The BMD difference of lumbar spine and wards area in combination therapy group was higher than Alendronate group after 6 months (*P* < 0.01) [46].

### Adverse effects of Qianggu Capsule

Six trials reported ADRs of Qianggu Capsule used alone [38–40, 42, 43, 45]. Three patients (3/41, 7.32%) with constipation [38] and 2 patients (2/32, 6.25%) with mild constipation [42] were found in Qianggu Capsule group. Zhao et al. reported that 3 patients (3/34, 8.82%) with constipation were identified in Qianggu Capsule group, whereas 3 patients (3/35, 8.57%) with uncomfortable hepatic region, 2 patients (2/35, 5.71%) with cutaneous pruritus, and 3 patients (2/35, 5.71%) with colporrhagia in livial group [39]. Xia et al. found that 2 patients (2/29, 6.90%) with constipation or dry mouth in Qianggu Capsule group, 1 patient (1/29, 3.45%) with constipation in the control group [40]. Similarly, Wang et al. reported 2 cases (2/28, 7.14%) with constipation and 1 case (1/28, 3.57%) with dry mouth in Qianggu Capsule group [43]. The study conducted by Gao et al. showed that 12 cases (12/64, 18.75%) with mild constipation, 15 cases (15/64, 23.44%) with dry mouth, and 18 cases (18/64, 28.13%) with lower rhythm of the heart in Qianggu Capsule group, while 9 cases (9/64, 14.06%) with loss of appetite, headache, vomit and 6 cases with higher blood calcium levels in the control group [45]. Only 1 trial observed the ADR of combination therapy [46]. The result demonstrated that 6 cases (6/40, 15%) with nausea in combination therapy group and 3 cases (3/40, 7.5%) with nausea in Alendronate group.

All of the ADRs were not severe and relieved without any treatment. Constipation and dry mouth were the most common ADRs in the usage of Qianggu Capsule.

### Quality of evidence

Based on the GRADE approach, low quality evidence (two trials, 208 participants) supported the Qianggu Capsule plus Caltrate D in improving BMD compared with Caltrate D; very quality evidence (three trials, 244 participants) supported the Qianggu Capsule in improving BMD compared withα-D3 capsule.

## Discussion

### Summary of the systematic review

More and more Chinese herbs have been historically used to treat bone metabolic diseases and known for anti-osteoporotic drugs [48–51]. The anti-osteoporosis effect of *Rhizoma Drynariae* and its extracts have attracted world-wide attention [52, 53]. Our systematic review is to assess the efficacy and safety of Qianggu Capsule (*Rhizoma Drynariae*) in osteoporosis therapy. The results of meta-analysis suggested that Qianggu Capsule plus Caltrate D was more effective than Caltrate D alone on lumbar spine, femoral neck and femoral great trochanter BMD [40, 47]. In addition, Qianggu Capsule had a more significant effect on femoral neck and femoral trochanteric BMD compared withα-D3 capsule [42, 43, 45]. No severe ADRs were found and the common ADRs could be improved promptly without special treatment.

So far, there is only one systematic review reporting Qianggu Capsule in treating POP [54]. Compared with previously reported review, our study strictly followed the PRISMA statement and added more randomised controlled trials. Secondly, the control groups were limited to be no treatment, placebo or conventional therapy. As well, for many complementary and alternative treatments there were not enough information about their efficacy and safety. So the alternative interventions were not enrolled as controls. Thirdly, we also summarized and analyzed the objective quantized outcomes, including bone formation and resorption markers.

### Recommendation on the Efficacy evaluation of Qianggu Capsule in the treatment of POP

In our study, definite conclusions could not be drawn in some subgroups because of the limited trials [38, 39, 41, 44, 46]. The meta-analysis was performed according to the homogeneity of the trials. Based on the current data, osteoporotic-fractures, quality of life and the related symptoms were not designed or evaluated in the included trials. BMD and metabolic markers were the most frequently reported outcomes. However, the results of Meta-analysis across trials were hampered by the high risk of bias, inconsistent result in some analysis, and small sample sizes (<400) on the basis of the GRADE approach. Eventually levels of quality of evidence were evaluated as low or very low. Thus, interpretation of these positive findings should be cautions.

On the other hand, the available meta-analysis did not confirm the efficacy for biochemical markers of bone turnover. The level of evidence was evaluated to be very low. One possible reason was the small sample sizes and short-term treatment. Meanwhile, some important bone turnover markers were not used for the diagnosis or evaluation in the primary studies. Accordingly, we suggest that serum procollagen type I amino-terminal propeptide (PINP) andβ-isomerised carboxy-terminal cross-linking telopeptide of type I collagen (CTX) be used as one of the important index, especially for the evaluation [55, 56].

### Limitation of this systematic review and direction for further clinical research

There are a number of methodological weaknesses in the previous studies. The majority of the included trials did not provide inadequate reporting of random method and allocation concealment. Only 1 trial used placebo-controlled design in our review [44]. Blinding is necessary to avoid detection bias. Randomized clinical trials without placebo design were likely to generate false positive results, such as the add-on design features (A + B versus B) [57]. It is difficult to evaluate the Qianggu Capsule absolute efficacy without a true placebo. Two trials did not report information on drop-out and withdraws [40, 44]. None of the included trials reported a pretrial estimation of sample size. All the studies were not large-scale randomized clinical trials. Since all the trials were published in Chinese journals, we could not exclude the potential publication bias.

Greater attention to methodological quality continues to be needed. In the future, large-sample and high-quality randomised, placebo-controlled trials should be conducted to further confirm the efficacy of Qianggu Capsule in treating POP. Since POP is a chronic metabolic disease, the effect of long-term treatment is a great concern of patients.

## Conclusions

Qianggu Capsule alone or Qianggu Capsule plus Caltrate D were beneficial for POP patients comparing to conventional interventions in improving BMD. Nevertheless, the evidence level was assessed to be low or very low according to GRADE approach. Therefore, the interpretation of that potential efficacy should be cautious, further research with strictly designed method is needed. Adverse outcomes of Qianggu Capsule mainly included constipation and dry mouth.

## Abbreviations

ADR: Adverse drug reaction
BMD: Bone mineral density
CBM: Chinese Biomedical Literature Database
CI: Confidence intervals
CNKI: Chinese national knowledge infrastructure
CTX: β-isomerised carboxy-terminal cross-linking telopeptide of type I collagen
MD: Mean difference
PINP: Procollagen type I amino-terminal propeptide
POP: Primary osteoporosis
RR: Risk ratio
SMD: Standardized mean difference
VIP: Chinese Scientific Journals Database
